# High diagnostic stability of confirmed migraine and confirmed tension-type headache according to the ICHD-3 beta in adolescents

**DOI:** 10.1186/1129-2377-15-36

**Published:** 2014-06-10

**Authors:** Lucia Albers, Andreas Straube, Mirjam N Landgraf, Florian Heinen, Rüdiger von Kries

**Affiliations:** 1 Division of Epidemiology, Institute of Social Paediatrics and Adolescent Medicine, Ludwig-Maximilians-University Munich, Haydnstr. 5, 80337 Munich, Germany; 2 Department of Neurology, Ludwig-Maximilians-University Munich, Munich, Germany; 3 Department of Paediatric Neurology and Developmental Medicine, Dr. von Hauner Children’s Hospital, Ludwig-Maximilians-University Munich, Munich, Germany

**Keywords:** Headache, Migraine, Tension-type headache, Adolescents, Stability, Tracking, Headache type, Probable diagnosis, Confirmed diagnosis

## Abstract

**Background:**

Stable headache diagnosis classification is a prerequisite for identification of headache type specific risk factors. Does the stability of a headache diagnosis over time vary between migraine and tension-type headache (TTH)? Are there differences in diagnosis stability between a probable and a definite headache diagnosis?

**Findings:**

In a sample of 783 students (ages 12 to 18 years) participating in a headache intervention study in greater Munich, the stability of headache classification according to the International Classification of Headache Disorder - third edition (beta version) (ICHD-3 beta) after a follow-up of 7 months was examined. Differences in stability of probable or definite migraine and probable or definite TTH were assessed. The stability of the headache diagnosis was assessed as predictive value of headache diagnosis with regard to confirmation of the headache type using the same diagnostic instrument 7 months later. Predictive values with 95% confidence intervals (CI) are reported.

Of students with initial migraine, a diagnosis of migraine was confirmed in 65.71% of students after 7 months (95%-CI [59.40-71.64]). A clear distinction between probable (44.71%, 95%-CI [33.91-53.89]) and confirmed diagnosis (76.88% 95%-CI [69.56-83.17]) of migraine was observed. For TTH the predictive value was 62.66% (95%-CI [57.07-68.01]) overall with a lower stability for probable (46.10%, 95%-CI [37.68-54.69]) compared to the confirmed diagnosis (69.71%, 95%-CI [23.58-37.67]).

**Conclusion:**

While confirmed migraine and confirmed TTH diagnoses seem stable over time, stability of a probable diagnosis for either headache type was lower.

**Trial registration:**

The trial was registered at the German Clinical Trial Register with the ID DRKS00003308.

## Introduction

Headache is a common health complaint in children and adolescents. A recent review reporting on 64 cross-sectional studies from the last 25 years from 32 different countries and including a total of 227,249 children and adolescents estimated an overall mean prevalence of headache of 54.4% (95%-CI [43.1;65.8]) and an overall mean prevalence of migraine of 9.1% (95%-CI [7.1-11.1]) [1]. Correct classification of the headache type is a prerequisite for targeted treatment [2]. In face of the high prevalence of headache in children and adolescents, prevention of headache is a major public health challenge. Epidemiological studies are required for the identification of appropriate preventive interventions. In these studies classification of headache types often cannot be based on an assessment by a physician. While headache in general appears to be a stable trait over time [3], some fluctuation regarding the type of headache has been reported in clinical cohorts [4-6], where headache classification was based on physicians’ diagnoses, as well as in epidemiological cohorts, where classification was based on questionnaires according to the International Classification of Headache Disorder [7-10].

Potential causes for these fluctuations in reported headache types are: A) Presence of genuinely two different types of headache in a person with different prevailing symptoms over time, of which only the dominant symptoms determine diagnostic classification. B) Genuine Change of headache type over time. C) Poor reliability of the questionnaire or the physicians’ diagnosis. D) Poor validity of the questionnaire. In the setting of a cluster randomised trial, we had the opportunity to assess diagnostic stability of probable and confirmed TTH and migraine diagnoses over a seven-month period: How stable is the ICHD classification of Migraine and TTH? Does the stability vary differently between confirmed and probable migraine or TTH?

## Findings

### Population

Our study population consists of 783 students recruited for the headache intervention study MUKIS (acronym for Münchner Untersuchung zu Kopfschmerzen bei Gymnasiasten – Interventionsstudie). MUKIS is a two-armed, prospective intervention study consisting of a baseline inquiry followed by an hour long in-class headache prevention lesson focusing on preventable risk factors for headache, as well as a follow-up inquiry approximately 7 months after the intervention. 12 grammar schools participated in the study. Participants were restricted to students of the 8th, 9th and 10th grades. Inclusion criteria for the present analysis was presence of headache (in the preceding 6 months) both at baseline and at follow-up (N = 783).

The mean age of the included students was 14.4 (range: 12–18 years) with a slight predominance of girls (62%, N = 489).

The study was approved by the Data Safety Officer and the Ethics Committee of the Medical Faculty of the Ludwig-Maximilians-University Munich and the Bavarian Ministry for Teaching and Culture. Parents and students (>14 years of age) gave written consent to participate in the study.

### Assessment of headache and clinical characteristics

Headache characteristics and headache type were assessed using a validated pain questionnaire for children and adolescents. Specific questions were added to the questionnaire to further classify headache subtype as migraine or tension-type headache (TTH) according the classification of the International Headache Society [11]. These additional questions provided all items necessary for diagnosis of TTH and migraine (both probable and confirmed diagnosis) according to the ICHD-3 beta version [12]. Frequency (days with headache) and intensity (on a 10 point visual analogue scale: mild headache intensity 1–3, moderate headache intensity 4–7 and severe headache intensity 7–10) in the preceding three months was assessed. For the classification of migraine, a headache duration of 4-72 h was required. Subjects fulfilling the criteria for confirmed migraine or confirmed TTH were labeled as such irrespective of potential symptoms for probable migraine or probable TTH. Individuals with both probable migraine and probable TTH criteria were given a combined diagnosis of migraine plus TTH. All subjects with headache that did not match any of these classifications for primary headache were considered to have miscellaneous headache (MiscH).

### Statistical analysis

The stability of headache diagnosis was examined by evaluating how well the initial headache diagnosis predicted the headache type seven months later, using the same diagnostic instrument. The frequencies of shifting classification of initial headache type at baseline to follow-up were calculated. Differences in stability between probable or definite migraine respectively TTH were assessed using Chi-Square statistics. A stable diagnosis of either migraine or TTH was assumed if adolescents with migraine at baseline had migraine or migraine plus TTH at follow-up, and respectively if adolescents with TTH at baseline had TTH or migraine plus TTH at follow-up.

### Study results

Diagnostic classification of headache did not change significantly for any of the categories in relation to the intervention (Migraine: p = 0.53, TTH: p = 0.97, Migraine + TTH: p = 0.45, MiscH: p = 0.55). Therefore merging the intervention and control groups to assess the stability of headache classification over time was justifiable.

**Figure 1:**
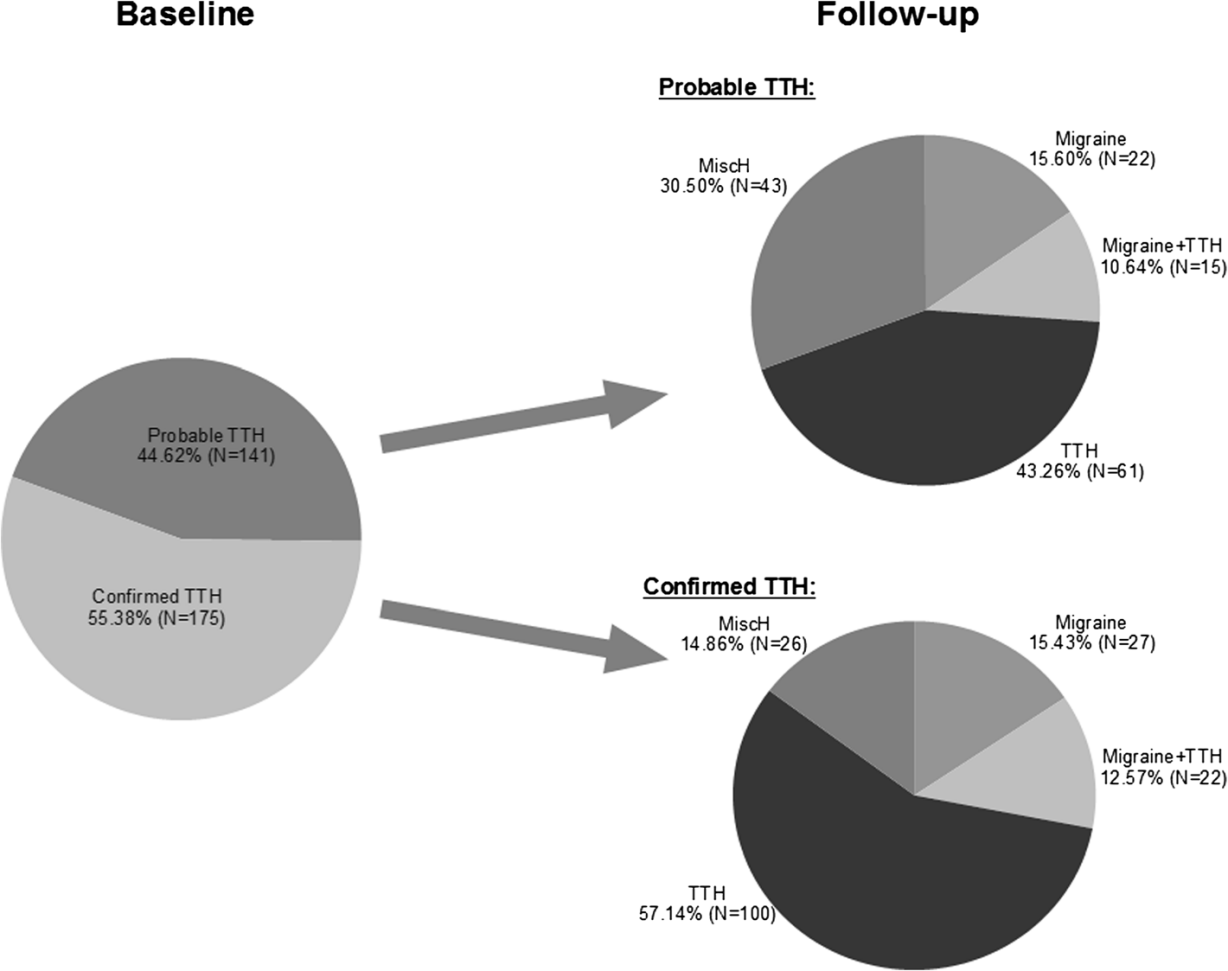
Development of headache type in 316 TTH patients after a follow-up of 7 months.

**Figure 2:**
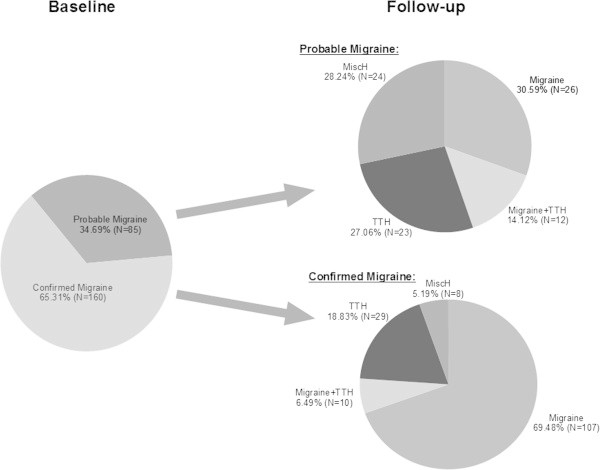
Development of headache type in 245 migraine patients after a follow-up of 7 months.

The overall stability of classification of migraine and TTH were both moderate (Table 1): On average, 60% of the adolescents with persistent headache classified as migraine or TTH had TTH or migraine or a combined diagnosis at follow-up.Considerable proportions of the initial TTH and migraine diagnoses were probable: 44.62% for TTH and 34.69% for migraine (Figures 1 and 2). The stability of an initially confirmed TTH diagnosis (N = 175) was clearly better than for an initially probable TTH (N = 141) (Figure 1). In migraine these differences were even more pronounced (p < 0.001): 76.88% adolescents with a confirmed migraine initially (N = 160) remained in the migraine or migraine plus TTH category seven months later (95%-CI [69.88-83.17]) as compared to only 44.71% (95%-CI [33.91-53.89]) of the adolescents initially in the probable migraine group (N = 85) (Figure 2).

**Table 1:** Shifting of headache diagnoses from baseline to follow-up in 783 students with headache

		**Follow-up**			
		**Migraine**	**TTH**	**Mig + TTH**	**MiscH**
		**%**			
		**(N)**			
**Baseline**	**Migraine**	54.29	21.22	11.43	13.06
	**N = 245**	(133)	(52)	(28)	(32)
	**TTH**	15.51	50.95	11.71	21.84
	**N = 316**	(49)	(161)	(37)	(69)
	**Mig + TTH**	36.84	26.32	22.11	14.74
	**N = 95**	(35)	(25)	(21)	(14)
	**MiscH**	13.39	33.07	8.66	44.88
	**N = 127**	(17)	(42)	(11)	(57)

The mean of number of days with headache in the preceding three months was 8.74 and only 13.71% reported mild headache. 5.14% of the students reported taking analgesics for every headache attack, 11.95% in nearly every headache attack, 14.78% sometimes, 22.88% rarely and 45.24% never. Possible medication overuse headache appeared possible in 1.28% of the adolescents at baseline (at least 15 headache attacks per month for the last three months treated with analgesics).

## Discussion

After a follow-up period of seven months, the stability of a confirmed diagnosis of either migraine or TTH in adolescents was high. Stability of diagnosis was lower for those with a probable diagnosis initially.

Compared to other studies, the diagnosis stability found in this study for both migraine and TTH was comparatively low [4-7]. A potential explanation for this might be a different proportion of individuals with confirmed versus probable migraine in different study populations. While most studies do not address this issue, two studies point to a higher stability of the confirmed diagnosis of migraine [4,7]. Our data are the first to show a significantly better diagnosis stability of confirmed migraine diagnosis as indicated by clearly disjunctive 95% confidence intervals.

The target of many headache intervention programs is to avoid risk factors for headache. Some of these risk factors are specific for certain headache types; for example, smoking and coffee consumption are strongly associated with migraine but not with TTH [13-16]. Thus in order to undertake headache-specific interventions, it is important to first understand the stability of a headache diagnosis.

Although the questionnaire used was not entirely validated parts of it had been [17,18]. The extension of the validated questionnaire was necessary in order to include all questions required to use the criteria of the ICHD-3 beta. Similar extensions have previously been used in other studies [11,19,20]. These extended versions have been shown to produce reasonably prevalence of headache and headache types. And although diagnosis of headache in children and adolescents is more difficult than in adults, good sensitivities of between 60-70% for similar screening questionnaires according to the ICHD-II were found (with the diagnosis of a headache specialist as gold standard) [21,22]. A high specificity (100%) for ICHD-II diagnosis of migraine as compared to an extensive interview by a headache specialist has been identified [21]. The Cohen’s kappa coefficient was 0.66 denoting a good level of agreement between both analyses.

Our study provides valid prevalence estimates for different headache types in grammar school students in Munich since all students in class at the first assessment answered the questionnaire. Follow-up was not related to type of headache, and thus precludes attrition bias. Potential causes for poor stability of diagnosis could be [2]: focus on only one headache type in the questionnaires thus ignoring potential occurrence of two headache types in one person (which is potentially relevant if the prevailing symptoms change over time); emergence of a new headache type (which is possible in our data); or poor reliability (unlikely as fair reliability of the questionnaire was recently demonstrated in structured interviews based on the International Headache Society (IHS) classification [23]). It is possible that the comparability of a self-administered questionnaire and a semi-structured interview may be limited, but from other fields of research there is some indication that the results of a self-administered questionnaire and structured interview might differ only marginally [24].

Although the questionnaire was self-administered, the students had the opportunity to ask questions for clarification within the classroom setting. A physician or a psychologist knowledgeable about the content of the questionnaire was present in the classroom.

A limitation of the study might be that we had no opportunity to identify secondary headache types. We are less concerned about this, however, since the average number of headache episodes in the last three months was only 8.7, suggesting that mild episodic secondary headache is unlikely to account for bias and only 14% of the study population reported having mild headache. Medication overuse headache (MoH) was not assessed in the questionnaire, but according to the review of Stovner and Andree only 1-2% of the European population suffer from MoH [25]. In our baseline data, only 1.28% of the adolescents report to have at least 15 headache attacks per month for the last 3 months and take analgesics for every or nearly every headache attack, therefore few of our headache cases are likely to be related to MoH.

A further limitation of our analysis might be the use of data from both arms of an intervention study for comparison of baseline and follow-up data. Since no significant difference could be found regarding diagnosis stability between control and intervention group such bias appears unlikely.

## Conclusion

Fair stability of confirmed migraine and confirmed tension-type headache was found in our study, whereas stability of probable migraine diagnosis and probable TTH diagnosis proved considerably lower.

## Abbreviations

TTH: Tension-type headache; Mig + TTH: Migraine plus tension-type headache; MiscH: Miscellaneous headache; MoH: Medication overuse headache; OR: Odds ratio; CI: Confidence interval; ICHD: International classification of headache disorder.

## Competing interests

The authors declare that they have no competing interests.

## Authors’ contributions

LA carried out the initial analyses, drafted the initial manuscript, and approved the final manuscript as submitted. FH, AS, MNL, RvK conceptualized and designed the study, revised the manuscript and approved the final manuscript as submitted.
